# Lightweight Cross-Modal Information Mutual Reinforcement Network for RGB-T Salient Object Detection

**DOI:** 10.3390/e26020130

**Published:** 2024-01-31

**Authors:** Chengtao Lv, Bin Wan, Xiaofei Zhou, Yaoqi Sun, Jiyong Zhang, Chenggang Yan

**Affiliations:** 1 School of Automation, Hangzhou Dianzi University, Hangzhou 310018, China; chengtaolv@outlook.com (C.L.); wanbinxueshu@icloud.com (B.W.); syq@hdu.edu.cn (Y.S.); jzhang@hdu.edu.cn (J.Z.); 2 Lishui Institute, Hangzhou Dianzi University, Lishui 323000, China

**Keywords:** salient object detection, RGB-T, lightweight, mutual reinforcement, multiscale information

## Abstract

RGB-T salient object detection (SOD) has made significant progress in recent years. However, most existing works are based on heavy models, which are not applicable to mobile devices. Additionally, there is still room for improvement in the design of cross-modal feature fusion and cross-level feature fusion. To address these issues, we propose a lightweight cross-modal information mutual reinforcement network for RGB-T SOD. Our network consists of a lightweight encoder, the cross-modal information mutual reinforcement (CMIMR) module, and the semantic-information-guided fusion (SIGF) module. To reduce the computational cost and the number of parameters, we employ the lightweight module in both the encoder and decoder. Furthermore, to fuse the complementary information between two-modal features, we design the CMIMR module to enhance the two-modal features. This module effectively refines the two-modal features by absorbing previous-level semantic information and inter-modal complementary information. In addition, to fuse the cross-level feature and detect multiscale salient objects, we design the SIGF module, which effectively suppresses the background noisy information in low-level features and extracts multiscale information. We conduct extensive experiments on three RGB-T datasets, and our method achieves competitive performance compared to the other 15 state-of-the-art methods.

## 1. Introduction

Salient object detection (SOD) is a computer vision technique that segments the most-visually interesting objects from an image, mimicking attention mechanisms. It is important to note that SOD differs from object detection tasks that aim to predict object bounding boxes. SOD has been employed as a preprocessing step in many computer vision tasks, such as image fusion [1], perceptual video coding [2], compressed video sensing [3], image quality assessment [4], and so on.

Traditional methods for RGB SOD were initially proposed, but they could not achieve optimal performance. With the advent of CNNs [5] and U-Nets [6], deep-learning-based methods became popular in SOD. For example, multiscale information was extracted in PoolNet [7] and MINet [8]. The edge feature was generated and supplemented to the object feature in EGNet [9] and EMFINet [10]. Later, depth maps were introduced in SOD, which is called RGB-D SOD. In this field, the depth-enhanced module [11] was designed to fuse two-modal features. However, the RGB-D dataset still has some shortcomings. Some depth maps are not accurate due to the limitations of the acquisition equipment. Researchers turned to introducing thermal infrared images into SOD, called RGB-T SOD.

**Figure 1:**
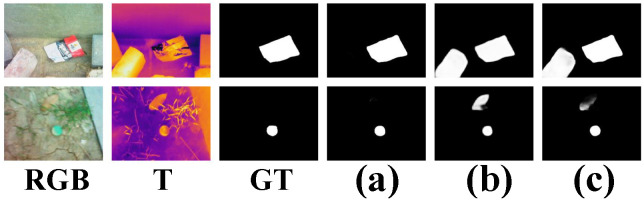
Some examples of RGB-T datasets. (**a**) Ours. (**b**) PCNet. (**c**) TAGF.

RGB-T SOD has seen significant progress in recent years. For example, CBAM [12] is employed in [13] to fuse two-modal features. To capture multiscale information, FAM module is employed in [13], and the SGCU module is designed in CSRNet [14]. Despite their outstanding efforts in RGB-T SOD, there are still some problems that need to be addressed. Most of the existing works are based on a heavy model, which is unsuitable for mobile devices. Besides, there is still room for research on effectively integrating the complementary information between two-modal features. Figure 1 shows some examples where PCNet [15] and TAGF [16] cannot present the detection results well. Another problem is how to fuse two-level features and explore multiscale information during the decoding stage.

Based on the aforementioned discussions, we propose a lightweight network for RGB-T SOD. Specifically, we employ the lightweight backbone MobileNet-V2 [17] in the encoder and the depth-separable convolution [18] in the decoder. To address the problem of two-modal feature fusion, we introduce the CMIMR module. We enhance two-modal features by transferring semantic information into them using the previous-level decoded feature. After this enhancement, we mutually reinforce two-modal features by communicating complementary information between them. Additionally, we design the SIGF module to aggregate two-level features and explore multiscale information during the decoding stage. Unlike RFB [11,19] and FAM [7], we employ the visual attention block (VAB) [20] to explore the multiscale information of the fused feature in the decoder.

Our main contributions are summarized as follows:

1. We propose a lightweight cross-modal information mutual reinforcement network for RGB-T salient object detection. Our network comprises a lightweight encoder, the cross-modal information mutual reinforcement (CMIMR) module, and the semantic-information-guided fusion (SIGF) module.
2. To fuse complementary information between two-modal features, we introduce the CMIMR module, which effectively refines the two-modal features.
3. Extensive experiments conducted on three RGB-T datasets demonstrate the effectiveness of our method.

## 2. Related Works

### Salient Object Detection

Numerous works have been proposed for SOD [21,22,23]. Initially, prior knowledge and manually designed features [24] were employed. With the advent of deep learning, CNN-based methods have made significant strides. For instance, many methods have attempted to capture multiscale information in images (RFB [19,25] and FAM [7]). Additionally, many works have focused on refining the edge details of salient objects [9,26,27]. Furthermore, several lightweight methods have been proposed to adapt to mobile devices [28,29]. While these methods have made great progress in RGB SOD, they do not perform as well when the RGB image has cluttered backgrounds, low contrast, and object occlusion.

RGB-D SOD is a technique that uses depth maps to provide complementary information to RGB images. To fuse two-modal features, several methods have been proposed, including the depth-enhanced module [11], selective self-mutual attention [30], the cross-modal depth-weighted combination block [31], the dynamic selective module [32], the cross-modal information exchange module [33], the feature-enhanced module [34], the cross-modal disentanglement module [35], the unified cross dual-attention module [36], and inverted bottleneck cross-modality fusion [37]. Despite the progress made by RGB-D SOD, it performs poorly on low-quality examples, where some depth maps are inaccurate due to the limitations of the acquisition equipment.

In addition to depth maps, thermal infrared images have been employed to provide complementary information to RGB images, which is called RGB-T SOD. Many works have made efforts in this area [38,39]. To fuse two-modal features, several methods have been proposed, including CBAM [12,13], the complementary weighting module [40], the cross-modal multi-stage fusion module [41], the multi-modal interactive attention unit [42], the effective cross-modality fusion module [43], the semantic constraint provider [44], the modality difference reduction module [45], the spatial complementary fusion module [46], and the cross-modal interaction module [15]. To fuse two-level features during the decoding stage, the FAM module [13] and interactive decoders [47] were proposed. Additionally, lightweight networks [14,48] have been proposed to meet the requirements of mobile devices.

## 3. Methodology

### 3.1. Architecture Overview

**Figure 2:**
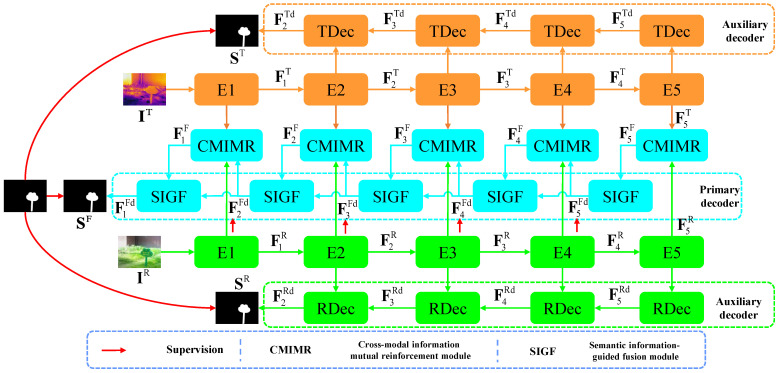
Overall architecture of our lightweight cross-modal information mutual reinforcement network for RGB-T salient object detection. ‘E1∼E5’ are the five modules of the encoder. ‘TDec’ and ‘RDec’ are the decoder modules of the auxiliary decoder. ‘CMIMR’ is the cross-modal information mutual reinforcement module. ‘SIGF’ is the semantic-information-guided fusion module.

We present the overall architecture of our method in Figure 2, which is a typical encoder–decoder structure. In the encoder part, we adopted the lightweight MobileNet-V2 (E1∼E5) [17] as the backbone to extract five-level features ${\left\{F_{i}^{R},F_{i}^{T}\right\}}_{i=1,\ldots ,5}$ for the two-modal inputs, respectively. To explore the complementary information between the two-modal features, we designed the cross-modal information mutual reinforcement module to fuse the two-modal features. To detect multiscale objects and fuse the two-level features, we designed the semantic-information-guided fusion module to suppress interfering information and explore multiscale information. Additionally, we employed two auxiliary decoder branches. On the one hand, this guides the two-modal encoders to extract modality-specific information [49] for the two-modal inputs, which helps to make the feature learning process more stable. On the other hand, this provides supplementary information in terms of single-channel saliency features. The decoder modules of the two auxiliary decoder branches are equipped with a simple structure, namely concatenation followed by 3×3 depth-separable convolution (DSConv) [18]. Finally, the 1×1 convolution is applied on three decoded features, resulting in three single-channel saliency features F1FdS, F2TdS, and F2RdS. After that, the sigmoid activation function is applied to obtain saliency maps $S^{F}$, $S^{T}$, and $S^{R}$. To fuse the complementary information between the three decoder branches, we summed the three single-channel saliency features and applied the sigmoid function to obtain the saliency map $S^{\mathrm{test}}$ during the testing stage. The above processes can be formulated as follows:

(1) F1FdS=Conv1×1F1FdF2TdS=Conv1×1F2TdF2RdS=Conv1×1F2Rd,

(2) SF=σF1FdSST=σF2TdSSR=σF2RdSStest=σF1FdS+F2TdS+F2RdS,

where $Conv_{1\times 1}$ means the 1×1 convolution and σ is the sigmoid function, which maps the single-channel saliency feature to the saliency map. $F_{1}^{\mathrm{Fd}}$, $F_{2}^{\mathrm{Td}}$, and $F_{2}^{\mathrm{Rd}}$ are the output features of the primary decoder and two auxiliary decoders.

### 3.2. Cross-Modal Information Mutual Reinforcement Module

Fusing complementary information between two-modal features is an essential question for RGB-T SOD. Two-modal features often contain noisy and inconsistent information, which can hinder the learning process of the saliency features. To address these issues, we designed the CMIMR module to suppress noisy information in the two-modal features and mutually supply valuable information.

**Figure 3:**
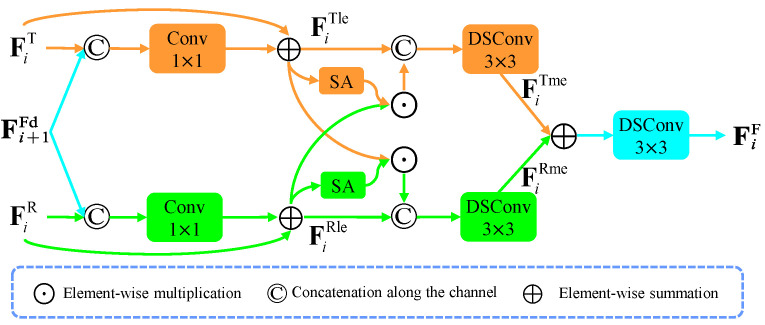
Architecture of the cross-modal information mutual reinforcement (CMIMR) module. ‘Conv1×1’ is the 1×1 convolution. ‘SA’ is the spatial attention. ‘DSConv3×3’ is the depth-separable convolution with the 3×3 convolution kernel.

The structure of the CMIMR module is illustrated in Figure 3. Specifically, we used the previous-level decoded feature, which contains accurate semantic and location information, to enhance the two-modal features by the concatenation–convolution operation, respectively. This guides the two-modal features to concentrate more on valuable information and alleviate background noise. However, this enhancement operation may weaken the beneficial information in the two-modal features. To address this issue, we added residual connections to the two-modal enhanced features. This process can be described as follows:

(3) $$\left\{\begin{array}{c} F_{i}^{\mathrm{Tle}}=F_{i}^{T}\;\\ F_{i}^{\mathrm{Rle}}=F_{i}^{R} \end{array}\right.i=5,$$

(4) $$\left\{\begin{array}{c} F_{i}^{\mathrm{Tle}}=F_{i}^{T}⊕Conv_{1\times 1}\left(\left[F_{i}^{T},Up_{\times 2}\left(F_{i+1}^{\mathrm{Fd}}\right)\right]\right)\;\\ F_{i}^{\mathrm{Rle}}=F_{i}^{R}⊕Conv_{1\times 1}\left(\left[F_{i}^{R},Up_{\times 2}\left(F_{i+1}^{\mathrm{Fd}}\right)\right]\right) \end{array}\right.i=1,\ldots ,4,$$

where ⊕ means elementwise summation and $Conv_{1\times 1}$ is the 1×1 convolution block consisting of the 1×1 convolution layer, and a batch normalization layer. [·,·] denotes concatenating two features along the channel dimension. $Up_{\times 2}$ means 2-times bilinear upsampling. $F_{i}^{T}$ and $F_{i}^{R}$ are the encoder features of the thermal image and RGB image at the *i*th-level. $F_{i}^{\mathrm{Tle}}$ and $F_{i}^{\mathrm{Rle}}$ are the previous-level information-enhanced two-modal features. $F_{i+1}^{\mathrm{Fd}}$ is the decoded feature at the (i+1)th level. The semantic and location information from the previous-level decoded features help suppress noisy information in the two-modal features, which facilitates the exploration of complementary information in the subsequent process.

After the aforementioned enhancement, we further exchanged the complementary information between the two-modal features. Since two-modal features contain both complementary and misleading information, directly concatenating them together can harm the appropriate fusion. Taking the RGB feature as an example, we intended to utilize the thermal feature to enhance it. Considering that spatial attention [50] can adaptively highlight regions of interest and filter the noisy information, we utilized the spatial attention map of the RGB feature to filter misleading information in the thermal features. This is because we wanted to preserve valuable information in the thermal feature, which is complementary to the RGB feature. After that, we concatenated the spatial-attention-filtered thermal feature with the RGB feature to supplement beneficial information into the RGB feature. Through this operation, the complementary information in the thermal feature can adaptively flow into the RGB feature, thereby obtaining a cross-modal information-enhanced RGB feature. The enhancement process for the thermal feature is similar to that of the RGB feature. Finally, we combined the two-modal enhanced features by elementwise summation to aggregate them:

(5) $$\left\{\begin{array}{c} F_{i}^{\mathrm{Tme}}=DSConv_{3\times 3}\left(\left[F_{i}^{\mathrm{Tle}},SA\left(F_{i}^{\mathrm{Tle}}\right)⊙F_{i}^{\mathrm{Rle}}\right]\right)\;\\ F_{i}^{\mathrm{Rme}}=DSConv_{3\times 3}\left(\left[F_{i}^{\mathrm{Rle}},SA\left(F_{i}^{\mathrm{Rle}}\right)⊙F_{i}^{\mathrm{Tle}}\right]\right)\;\\ F_{i}^{F}=DSConv_{3\times 3}\left(F_{i}^{\mathrm{Tme}}⊕F_{i}^{\mathrm{Rme}}\right) \end{array}\right.i=1,\ldots ,5,$$

where $DSConv_{3\times 3}$ is the 3×3 DSConv layer [18], ⊙ represents the elementwise multiplication operation, and SA denotes the spatial attention [50]. $F_{i}^{\mathrm{Tme}}$ and $F_{i}^{\mathrm{Rme}}$ are cross-modal enhanced two-modal features. $F_{i}^{F}$ is the two-modal fused feature. In summary, the CMIMR module can effectively suppress background noise in two-modal features under the guidance of previous-level semantic information. Furthermore, it can supplement valuable information to each modal feature, which helps to effectively fuse the two-modal features.

### 3.3. Semantic-Information-Guided Fusion Module

How to design the two-level feature aggregation module during the decoding stage is a crucial question for SOD. It is related to whether we can recover the elaborate details of salient objects. Since low-level features contain much noisy information, directly concatenating them together will inevitably introduce disturbing information into the fused features. To rectify the noisy information in the low-level features, we transmitted the semantic information in the high-level feature into it. Besides, multiscale information is vital in SOD tasks. Salient objects in different scenes are of various sizes and shapes, but the ordinary 3×3 convolution cannot accurately detect these salient objects. Inspired by the great success of multiscale information-capture modules (e.g., RFB [7,11] and FAM [19]) in SOD, we employed the visual attention block (VAB) [20] to capture the multiscale features. The VAB was initially designed as a lightweight feature-extraction backbone for many visual tasks.

**Figure 4:**
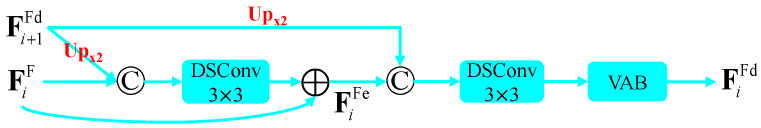
Architecture of the semantic-information-guided fusion (SIGF) module. ‘DSConv3×3’ is the depth-separable convolution with the 3×3 convolution kernel. ‘VAB’ is the visual attention block. ‘$Up_{\times 2}$’ is the two-times upsample.

The SIGF module structure is shown in Figure 4. Specifically, to suppress the background noisy information in the low-level feature, we utilized the high-level feature to refine the feature representation of the low-level feature. We concatenated the high-level feature into the low-level feature to enhance it. In the feature-enhancement process, valuable information in the low-level features may be diluted, so we introduced residual connections to preserve it. This process can be expressed as follows:

(6) $$F_{i}^{\mathrm{Fe}}=F_{i}^{F}⊕DSConv_{3\times 3}\left(\left[F_{i}^{F},Up_{\times 2}\left(F_{i+1}^{\mathrm{Fd}}\right)\right]\right)\;i=1,\ldots ,4,$$

where $F_{i}^{\mathrm{Fe}}$ is the semantic-information-enhanced feature. $F_{i+1}^{\mathrm{Fd}}$ is the decoded feature at the ${\left(i+1\right)}_{th}$ level. $F_{i}^{F}$ is the two-modal fused features. Then, to enable our method to detect salient objects of various sizes and shapes, we used the VAB to extract multiscale information contained in the fused features:

(7) $$F_{i}^{\mathrm{Fd}}=\left\{\begin{array}{cc} VAB\left(F_{i}^{F}\right) & i=5\\ VAB\left(DSConv_{3\times 3}\left(\left[F_{i}^{\mathrm{Fe}},Up_{\times 2}\left(F_{i+1}^{\mathrm{Fd}}\right)\right]\right)\right) & i=1,\ldots ,4 \end{array}\right.,$$

where VAB is the visual attention block [20]. $F_{i}^{\mathrm{Fd}}$ is the decoded feature at the *i*th level. The VAB consists of two parts: the large kernel attention (LKA) and feed-forward network (FFN) [51]. In the large kernel attention, the depth-separable convolution, depth-separable dilation convolution with dilation d, and a 1×1 convolution are successively stacked to capture multiscale information:

(8) $$\left\{\begin{array}{c} VAB\left(F\right)=FFN\left(LKA\left(F\right)\right)\;\\ LKA\left(F\right)=Conv_{1\times 1}\left(DSConv_{d}\left(DSConv\left(F\right)\right)\right)⊙F \end{array}\right.,$$

where $DSConv_{d}$ is the depth-separable convolution with dilation *d*. F stands for the feature being processed. In summary, our module can rectify noisy information in the low-level feature under the guidance of high-level accurate semantic information. Meanwhile, the VAB successfully extracts multiscale information, which is beneficial for detecting multiscale salient objects.

### 3.4. Loss Function

The deep supervision strategy [52] is adopted in our method. Specifically, the saliency predictions of deep features ${F_{i}^{\mathrm{Fd}}}_{\left(i=1,\ldots ,5\right)}$ are supervised, as shown in Figure 2. Additionally, the saliency predictions of two auxiliary decoders’ output features $F_{2}^{\mathrm{Td}}$, $F_{2}^{\mathrm{Rd}}$ are also supervised. The BCE loss [53] and IoU loss [54] are employed to calculate the losses between saliency predictions and the GT:

(9) $$\left\{\begin{array}{c} \begin{array}{ccc} \ell_{all} & = & \sum_{i=1}^{5}\frac{1}{2^{i-1}}\ell_{loss}\left(S_{i}^{F},G\right)+\ell_{loss}\left(S^{T},G\right)+\ell_{loss}\left(S^{R},G\right)\;\\ \ell_{loss} & = & \ell_{bce}+\ell_{IoU} \end{array} \end{array}\right.,$$

where $S_{i}^{F}$, $S^{T}$, and $S^{R}$ mean the saliency predictions of the deep features $F_{i}^{\mathrm{Fd}}$, $F_{2}^{\mathrm{Td}}$, and $F_{2}^{\mathrm{Rd}}$, respectively. G means the ground truth. $\ell_{bce}$ and $\ell_{IoU}$ mean the BCE loss and IoU loss, respectively.

## 4. Experiments

### 4.1. Experiment Settings

#### 4.1.1. Datasets

There are three RGB-T SOD datasets that have been widely employed in existing works: VT821 [55], VT1000 [56], and VT5000 [13]. VT821 consists of 821 manually registered RGB-T image pairs. VT1000 is composed of 1000 well-aligned RGB-T image pairs. VT5000 has 5000 RGB-T image pairs, containing complex scenes and diverse objects. Following the previous works’ setting [47], 2500 samples from VT5000 were selected as the training dataset. The other 2500 samples from VT5000 and all samples from VT821 and VT1000 served as the testing datasets. To avoid overfitting, the training dataset was augmented by random flipping and random rotation [11].

#### 4.1.2. Implementation Details

The model was trained on a GeForce RTX 2080 Ti (11GB memory). The Pytorch framework was employed in the code implementation. The encoders were initialized with the pre-trained MobileNet-V2 [17], while the other parameters were initialized with the Kaiming uniform distribution [57]. The input image was resized to 224×224 for both the training and testing stages. The training epochs and batch size were set to 120 and 20, respectively. The Adam optimizer was employed to reduce the loss of our method. The learning rate was set to $1\times 10^{-4}$ and will decay to $1\times 10^{-5}$ after 90 epochs.

### 4.2. Evaluation Metrics

To compare the performance of our method with other methods, four numeric evaluation metrics were employed, the mean absolute error (M), F-measure ($F_{\beta }$) [58], E-measure ($E_{\xi }$) [59], and structure-measure ($S_{\alpha }$) [60]. Besides, the PR curve and F-measure curve are plotted to show their evaluation results.

#### 4.2.1. M

The mean absolute error M calculates the mean absolute error between the prediction value and the GT:

(10) $$M=\frac{1}{W\times H}\sum_{i=1}^{W}\sum_{j=1}^{H}\left|S\left(i,j\right)-G\left(i,j\right)\right|,$$

where G(i,j) and S(i,j) denote the ground truth and the saliency map, respectively.

#### 4.2.2. $F_{\beta }$

The F-measure $\left(F_{\beta }\right)$ is the weighted harmonic mean of the recall and precision, which is formulated as

(11) $$F_{\beta }=\frac{(1+\beta^{2})Precision\cdot Recall}{\beta^{2}\cdot Precision+Recall},$$

where $\beta^{2}$ was set to 0.3, referring to [58].

#### 4.2.3. $E_{\xi }$

The E-measure $\left(E_{\xi }\right)$ evaluates the global and local similarities between the ground truth and predictions:

(12) $$E_{\xi }=\frac{1}{W\times H}\sum_{i=1}^{W}\sum_{j=1}^{H}\varphi \left(S(i,j),G(i,j)\right),$$

where φ is the enhanced alignment matrix.

#### 4.2.4. $S_{\alpha }$

The structure-measure $\left(S_{\alpha }\right)$ evaluates the structural similarities of salient objects between the ground truth and predictions:

(13) $$S_{\alpha }=\alpha S_{o}+\left(1-\alpha \right)S_{r},$$

where $S_{r}$ and $S_{o}$ mean region-aware and object-aware structural similarity, respectively, and α was set to 0.5, referring to [60].

### 4.3. Comparisons with the SOTA Methods

To show the effectiveness of our method, we compared it with 15 SOTA methods, the RGB SOD methods BASNet [27], EGNet [9], and CPD [19] and the RGB-T SOD methods ADF [13], MIDD [47], MMNet [41], MIADPD [42], OSRNet [61], ECFFNet [43], PCNet [15], TAGF [16], UMINet [62], MGAI [63], APNet [64], CGFNet [65], CSRNet [14], and LSNet [48]. For a fair comparison, the saliency maps of all compared methods are either directly provided by the author or re-implemented by the official public code.

#### 4.3.1. Quantitative Comparison

**Figure 5:**
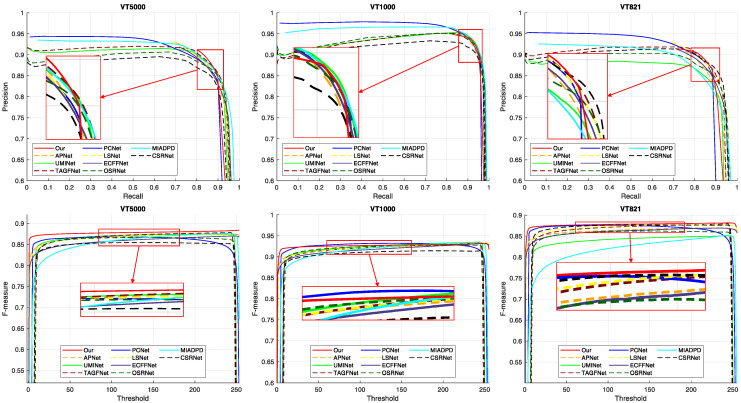
PR curves and F-measure curves of the compared methods on the RGB-T datasets.

We compared the performance of the heavy-model-based methods in Table 1 and the lightweight methods in Table 2. The PR and F-measure curves of the compared methods on the three RGB-T datasets are plotted in Figure 5. Our method outperformed most methods in terms of four metrics, except for $S_{\alpha }$, which was slightly inferior to the other methods. Compared to the heavy-model-based methods, as shown in Table 1, our method improved 6.9%, 2.0%, and 1.1% in terms of M, $F_{\beta }$, and $E_{\xi }$ on VT5000. Although our method was not as good as other methods in terms of $S_{\alpha }$, it requires only 6.1M parameters and 1.5G FLOP and can be easily applied to mobile devices. The inference speed of our method was mediocre on a professional GPU (GeForce RTX 2080 Ti, Santa Clara, CA, USA) with 34.9 FPS. However, given that the mobile devices only have access to the CPU, our method outperformed the other methods with 6.5 FPS (AMD Ryzen 7 5800H, Santa Clara, CA, USA). Besides, we compare our method with existing lightweight methods in Table 2. Our method outperformed the other methods on most metrics, except for $S_{\alpha }$ on VT1000 and VT821. Our method improved 12.5%, 2.3%, and 1.2% in terms of M, $F_{\beta }$, and $E_{\xi }$ on VT5000. Among the lightweight methods, the FLOP and FPS of our method were not as good as LSNet, but our method performed better. In addition, we plot the PR and F-measure curves in Figure 5 to visually compare the performance of all methods. We can see that the precision of our method was higher than other methods on VT5000 and VT821, when the recall was not very close to 1. The F-measure curves consider the trade-offs between precision and recall. We can see that our method obtained better F-measure scores on VT5000 and VT821. We evaluate the IoU and Dice scores of our method in Table 3 with reference to most image segmentation tasks. We can see that our method performed better on VT1000 than on VT5000 and VT821. Additionally, our method outperformed the compared method LSNet on all three datasets.

To demonstrate the significance of the performance improvement of our method, the *t*-test was performed. We retrained our method and obtained six sets of experiment results, shown in Table 4. Concretely, assuming the metrics $X∼N(\mu ,\sigma^{2})$, the test statistic was $t=\frac{\bar{X}-\mu_{0}}{S/\sqrt{n}}$, where $S^{2}$ is an unbiased estimate of $\sigma^{2}$. $\frac{\bar{X}-\mu_{0}}{S/\sqrt{n}}∼t\left(n-1\right)$. t(n−1) is the Student distribution with n−1 degrees of freedom. Therefore, the *t*-test was used in our hypothesis test. For the evaluation metric M, the left-sided test was performed, i.e., the $H_{0}$ hypothesis was that the M of our method was greater than that of the compared method. For the other five metrics $F_{\beta }$, $S_{\alpha }$, $E_{\xi }$, IoU, and Dice, the right-sided test was performed, i.e., the $H_{0}$ hypothesis was that the corresponding results of our method were less than those of the compared method. The *p*-value is reported in our *t*-test. Three significance levels α were used in our *t*-test, i.e., 0.01, 0.05, and 0.1. Generally speaking, if *p*-value ≤ 0.01, the test is highly significant. If 0.01 < *p*-value ≤ 0.05, the test is significant. If 0.05 < *p*-value ≤ 0.1, the test is not significant. If *p*-value > 0.1, then there is no reason to reject the $H_{0}$ hypothesis. As shown in Table 5, the *p*-value of our method was less than 0.01 for M, $F_{\beta }$, and $E_{\xi }$ on the three datasets, indicating that the *t*-test was highly significant.

#### 4.3.2. Qualitative Comparison

**Figure 6:**
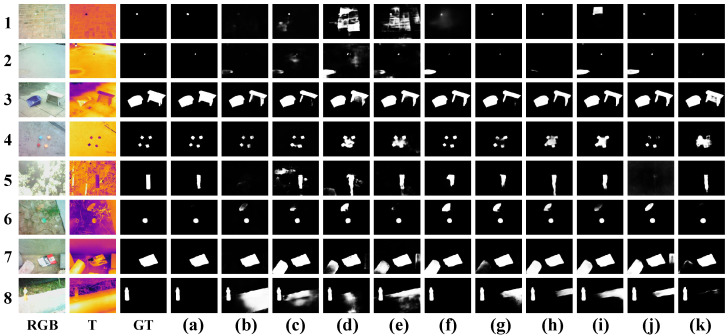
Visual comparisons with other methods. (**a**) Ours. (**b**) ADF. (**c**) MIDD. (**d**) MMNet. (**e**) MIADPD. (**f**) OSRNet. (**g**) ECFFNet. (**h**) PCNet. (**i**) TAGF. (**j**) UMINet. (**k**) APNet.

To demonstrate the effectiveness of our method, we also provide the visual comparisons with other methods in Figure 6. In this figure, the challenging scenes include small objects (1st and 2nd row), multiple objects (3rd and 4th row), a misleading RGB image (5th row), and misleading thermal images (6th, 7th, and 8th row). As seen in Figure 6, our method can detect salient objects better than other methods. For example, in the first and second rows, our method can accurately detect small salient objects, while other methods like MMNet and MIADPD failed in this case. In the third and fourth rows, our method can detect multiple objects in the scene, but the other methods performed poorly. In the fifth row, our method can detect the salient object effectively despite the low contrast in the RGB image, while the other methods were interfered with by the noisy information in the RGB image. In the sixth and seventh rows, the salient objects have apparent contrast in the RGB image, but are similar to other objects in the background in the thermal image. The thermal images provide misleading information, which can be easily solved by our method. In summary, our method can accurately overcome the challenges in these scenarios due to the better fusion of the complementary information between the two-modal features and multiscale information extraction.

### 4.4. Ablation Study

#### 4.4.1. Effectiveness of Cross-Modal Information Mutual Reinforcement Module

To demonstrate the effectiveness of the CMIMR module, we perform several ablation experiments in Table 6. First, we removed the CMIMR module, i.e., the two-modal features were directly concatenated followed by the 3×3 DSConv, referred to as *w*/*o* CMIMR. Compared with this variant, our method improved M and $F_{\beta }$ by 5.0% and 1.7% on VT5000, respectively. This suggests that our method can effectively fuse complementary information between two-modal features by enhancing them with the guidance information of the previous level and inter-modality. To demonstrate that the performance improvement of each module is significant, we perform *t*-test in Table 7. As shown in Table 7, the *p*-value of our method was less than 0.01 for all four metrics compared to the variant *w*/*o* CMIMR, so the test was highly significant. To demonstrate that the CMIMR outperformed the other modules that play the same role in existing methods, we replaced it with the two-modal feature fusion module in ADF [13], abbreviated as *w* ADF-TMF. Compared to this variant, our method improved the M and $F_{\beta }$ by 2.4% and 0.8% on VT5000, respectively. Compared to the variant *w* ADF-TMF, the *p*-value of our method was less than 0.01 for $F_{\beta }$ and $S_{\alpha }$ on VT5000, so the test was highly significant. This suggests that the design of the CMIMR module is sound.

**Figure 7:**
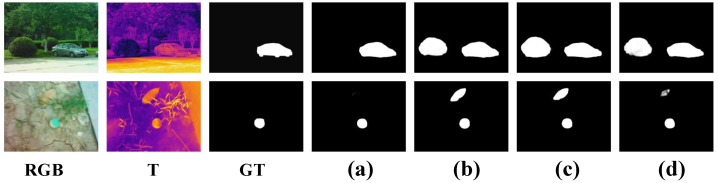
Visual comparisons with ablation experiments on the effectiveness of the CMIMR module. (**a**) Ours. (**b**) *w*/*o* CMIMR. (**c**) *w*/*o* PDFE. (**d**) *w*/*o* IMR.

Second, we removed the previous-level decoded feature enhancement, which is abbreviated as *w*/*o* PDFE, i.e., two-modal features are not enhanced by the previous-level decoded feature, but are directly fed into the cross-modal information mutual enhancement component of the CMIMR module. Compared to this variant, our method improved the M and $F_{\beta }$ by 2.1% and 0.8% on VT5000, respectively. Compared to the variant *w*/*o* PDFE, the *p*-value of our method was less than 0.01 for the $F_{\beta }$, $S_{\alpha }$, and $E_{\xi }$ on VT5000; therefore, the test was highly significant. This shows that the PDFE component is conducive to suppressing noisy information in two-modal features. Finally, we removed the cross-modal information mutual reinforcement component, which is abbreviated as *w*/*o* IMR, i.e., after the PDFE component, the two-modal features were fused by the concatenation–3×3 DSConv. Compared to this variant, our method improved the M and $F_{\beta }$ by 3.0% and 0.8% on VT5000, respectively. Compared to the variant *w*/*o* IMR, the *p*-value of our method was less than 0.01 for the $F_{\beta }$, $S_{\alpha }$, and $E_{\xi }$ on VT5000, so the test was highly significant. This suggests that the IMR component helps to transfer complementary information to each other and suppress the distracting information in each modality. We also show the saliency maps of the ablation experiments in Figure 7. In the first row, the holly is obvious in the RGB image, and other ablation variants mistook it for salient objects. In the second row, the potato in the thermal image is similar to the salient objects, and other ablation variants cannot distinguish it accurately. However, with the CMIMR module, our method can eliminate this misleading information. In conclusion, the CMIMR module can effectively fuse the complementary information between two-modal features and mitigate the adverse effects of distracting information.

#### 4.4.2. Effectiveness of Semantic-Information-Guided Fusion Module

To demonstrate the effectiveness of the semantic-information-guided fusion module, we conducted three ablation experiments. The results are shown in Table 6. First, we removed the SIGF module in our method, abbreviated as *w*/*o* SIGF, i.e., the two-level features were directly concatenated, followed by the 3×3 DSConv. Compared to this variant, our method improved the M and $F_{\beta }$ by 3.9% and 1.2% on VT5000, respectively. This demonstrates that the SIGF module is helpful in suppressing interfering information and exploring multiscale information. To demonstrate that the performance improvement of the SIGF module is significant, we perform the *t*-test in Table 7. Compared to the variant *w*/*o* SIGF, the *p*-value of our method was less than 0.01 for four metrics on VT5000, so the test was highly significant, except for the *p*-value, which was less than 0.05 for $S_{\alpha }$ on VT821, which was significant. To demonstrate that the SIGF module outperformed other the modules that play the same role in existing methods, we replaced it with the decoder module in ADF [13], abbreviated as *w* ADF-Decoder. Compared to this variant, our method improved the M and $F_{\beta }$ by 2.4% and 1.0% on VT5000, respectively. Compared to the variant *w* ADF-Decoder, the *p*-value of our method was less than 0.01 for $F_{\beta }$ on VT5000, so the test was highly significant. This suggests that the design of the SIGF module is sound.

**Figure 8:**
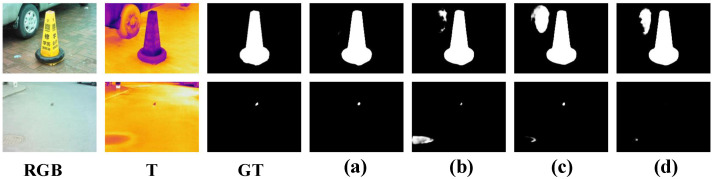
Visual comparisons with ablation experiments on the effectiveness of the SIGF module. (**a**) Ours. (**b**) *w*/*o* SIGF. (**c**) *w*/*o* SIE. (**d**) *w*/*o* VAB.

Second, we removed the previous-level semantic information enhancement in the SIGF module, which is abbreviated as *w*/*o* SIE, i.e., the previous-level semantic information enhancement was removed, and the two-level features were directly concatenated in the SIGF module. Compared with this variant, our method improved the M and $F_{\beta }$ by 1.8% and 0.7% on VT5000, respectively. This demonstrates that the SIE component helps to suppress interfering information. Compared to the variant *w*/*o* SIE, the *p*-value of our method was less than 0.05 for the $F_{\beta }$, $S_{\alpha }$, and $E_{\xi }$ on VT5000, so the test was significant. Next, we removed the VAB component in the SIGF module, which is abbreviated as *w*/*o* VAB, i.e., the VAB component was removed in the SIGF module, and the other components were retained. Compared to this variant, our method improved the M and $F_{\beta }$ by 2.7% and 0.8% on VT5000, respectively. This shows that the VAB is capable of capturing the multiscale information of salient objects. Compared to the variant *w*/*o* VAB, the *p*-value of our method was less than 0.01 for the $F_{\beta }$ and $S_{\alpha }$ on VT5000, so the test was highly significant. Besides, we also replaced the VAB in the SIGF module with the RFB and FAM, abbreviated as *w* SIGF-RFB and *w* SIGF-FAM, respectively. Compared to the RFB variant, our method improved the M and $F_{\beta }$ by 2.1% and 0.6% on VT5000, respectively. Compared to the variant *w* SIGF-RFB, the *p*-value of our method was less than 0.05 for the $F_{\beta }$ and $E_{\xi }$ on VT5000, so the test was significant. Compared to the FAM variant, our method improved the M and $F_{\beta }$ by 2.1% and 0.6% on VT5000, respectively. These two results indicate that the VAB slightly outperformed the RFB and FAM in capturing multiscale context information. We also show the visual comparisons of the ablation experiments in Figure 8. In the first row, the variants are disturbed by the tire. In the second row, other variants are unable to detect small objects. With the SIGF module, our method effectively addresses these challenges. In summary, the SIGF module can effectively suppress interfering information and capture multiscale information.

#### 4.4.3. Effectiveness of Hybrid Loss and Auxiliary Decoder

**Figure 9:**
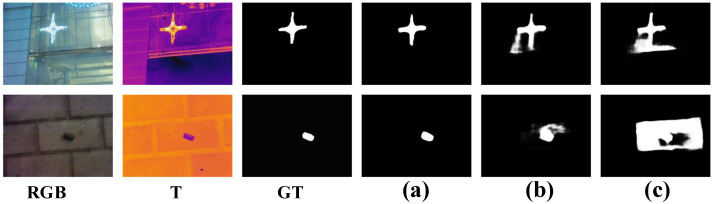
Visual comparisons with ablation experiments on the effectiveness of the IoU loss and auxiliary decoder. (**a**) Ours. (**b**) *w*/*o* IoU. (**c**) *w*/*o* AD.

To demonstrate the effectiveness of the hybrid loss and auxiliary decoder, we conducted two ablation experiments. The results are presented in Table 6. First, we removed the IoU loss, which is abbreviated as *w*/*o* IoU, i.e., only the BCE loss was employed in training our model. Compared to this variant, our method improved the M and $F_{\beta }$ by 3.0% and 1.4% on VT5000, respectively. Compared to the variant *w*/*o* IoU, the *p*-value of our method was less than 0.01 for the $F_{\beta }$ and $E_{\xi }$ on VT5000, so the test was highly significant. This demonstrates that the IoU loss is conducive to boosting the performance from the perspective of integral consistency. As shown in Figure 9b, the variant *w*/*o* IoU is susceptible to background noise. To demonstrate of the effectiveness of summing three single-channel saliency features, we employed three learnable parameters to weight them and, then, summed the weighted features, abbreviated as *w* LPW. Compared to this variant, our method improved the M and $F_{\beta }$ by 4.2% and 1.8% on VT5000, respectively. Compared to the variant *w* LPW, the *p*-value of our method was less than 0.01 for M, $F_{\beta }$, and $E_{\xi }$ on VT5000, so the test was highly significant. However, our method failed to perform in the $S_{\alpha }$, i.e., the learnable parameters can improve the $S_{\alpha }$, but it did not perform as well as our method on the other metrics. Besides, we also conducted an experiment on the summation of three saliency maps, abbreviated as $S^{F}$ + $S^{R}$ + $S^{T}$. The results were even worse than those only employing $S^{F}$. Compared to this variant, our method improved the M and $F_{\beta }$ by 20.1% and 10.6% on VT5000, respectively. Compared to the variant $S^{F}$ + $S^{R}$ + $S^{T}$, the *p*-value of our method was less than 0.01 for four metrics on VT5000, so the test was highly significant. This suggests that summing the three saliency maps together can have a detrimental effect. In Table 6, we also report the evaluation results of the three saliency maps, abbreviated as $S^{F}$, $S^{R}$, and $S^{T}$, respectively. Note that we wished to evaluate the contribution of the three saliency maps ($S^{F}$, $S^{R}$, and $S^{T}$) in the same setup as our full method, and therefore, the network parameters remained unchanged. The primary decoder saliency map $S^{F}$ was much better than the two auxiliary decoder saliency maps $S^{R}$ and $S^{T}$. Compared to the $S^{F}$, our method improved the M and $F_{\beta }$ by 1.8% and 0.8% on VT5000, respectively. This suggests that summing three single-channel saliency features can also provide beneficial information for $S^{F}$. Unfortunately, however, this strategy had an adverse effect on $S_{\alpha }$, reducing the $S_{\alpha }$ by 0.6% on VT5000.

We also conducted experiments only employing one modality as the input, abbreviated as RGB and T. That is, two auxiliary decoders were removed, the CMIMR module was removed, and no two-modal feature fusion were required since only one modality was used as the input. We input the RGB image and thermal image into the modified network separately. Then, the SIGF module was employed to decode the two-level features from top-to-bottom. Only employing the RGB image as the input was better than only employing the T image, but our method can greatly improve the results. Compared to the variant RGB, out method improved the M and $F_{\beta }$ by 23.4% and 4.4% on VT5000, respectively. Compared to the variant RGB, the *p*-value of our method was less than 0.01 for four metrics on VT5000, so the test was highly significant.

Besides, to demonstrate the necessity of two auxiliary decoders, we removed two auxiliary decoders, which is abbreviated as *w*/*o* AD, i.e., only the primary decoder was retained in our modified model. Compared to this variant, our method improved the M and $F_{\beta }$ by 10.8% and 2.0% on VT5000, respectively. Compared to the variant *w*/*o* AD, the *p*-value of our method was less than 0.01 for four metrics on VT5000, so the test was highly significant. This demonstrates that two auxiliary decoders can guide the two-modal encoders to extract modality-specific information and supplement valuable information at the single-channel saliency feature level. Unfortunately, the AD module did not perform well in all cases, but considering that it boosted most metrics, its failure cases in $S_{\alpha }$ are acceptable. Note that since the network structure was modified in these three cases (*w*/*o* AD, RGB, and T), we needed to retrain the network to obtain the saliency maps, which is a different experimental setup from the ablation experiments $S^{F}$, $S^{R}$, and $S^{T}$. As shown in Figure 9c, the variant *w*/*o* AD failed to guide two encoders to extract beneficial information. On the contrary, our entire model performed well in these cases.

### 4.5. Scalability on RGB-D Datasets

To demonstrate the scalability of our method, we retrained it on the RGB-D datasets. Following the settings in [66], we employed the 1485 images from NJU2K [67] and 700 images from NLPR [68] as the training datasets. The other parts of NJU2K, NLPR, and all images of SIP [66], STERE1000 [69] were taken as the testing datasets. Note that when testing on DUT [70], the extra 800 images from DUT were also taken as the training datasets, namely a total of 2985 images for training on DUT.

To demonstrate the effectiveness of our method, we compared it with 10 SOTA methods, S2MA [30], AFNet [71], ICNet [31], PSNet [72], DANet [73], DCMF [35], MoADNet [37], CFIDNet [34], HINet [33], and LSNet [48]. As shown in Table 8, our method improved 3.2% and 0.5% in terms of the M and $E_{\xi }$ on the NJU2K dataset. Besides, our method improved 0.8% and 0.9% in terms of the M and $F_{\beta }$ on the NLPR dataset. This demonstrates that our method has a preferable generalization ability on the RGB-D datasets. To demonstrate that the performance improvement of our method was significant, the *t*-test is performed in Table 9. We retrained our method and obtained six sets of experiment results. As shown in Table 9, compared to other methods, the *p*-value of M, $F_{\beta }$, and $E_{\xi }$ on NJU2K were less than 0.01; therefore, the *t*-test was highly significant. The *p*-value of M and $F_{\beta }$ on NLPR were less than 0.01; therefore, the test was highly significant.

## 5. Discussion

This paper further identifies three important issues in RGB-T SOD: two-modal feature fusion, two-level feature fusion, and the saliency information fusion of three decoder branches. It also provides feasible solutions to these issues, which researchers can use to make further improvements. Our method has three advantages. First, in the two-modal feature fusion, the supplementary information is retained and interfering information is filtered. Second, in the two-level feature fusion, the guidance of the semantic information helps to suppress noise information in the low-level features. Third, the auxiliary decoder can guide the two encoders to extract modality-specific information. However, there are limitations to our method. First, the summation of three single-channel saliency features improves other the metrics, but degrades the $S_{\alpha }$. Second, while the full CMIMR and SIGF bring significant improvements to our method, their subcomponents do not largely improve the metrics. We will further address these limitations in future work. There are several directions for future development in this field. First, boundary information should be taken into account to recover clearer boundaries of salient objects. Second, although existing methods have made great progress, the structure is complex and simpler, and more-effective solutions need to be explored. Finally, the solutions of two-modal feature fusion and two-level feature fusion need further improvement.

## 6. Conclusions

In this paper, we propose a lightweight cross-modal information mutual reinforcement network for RGB-T salient object detection. Our proposed method consists of the cross-modal information mutual reinforcement module and the semantic-information-guided fusion module. The former module fuses complementary information between two-modal features by enhancing them with semantic information of the previous-level decoded feature and the inter-modal complementary information. The latter module fuses the two-level features and mines the multiscale information from the deep features by rectifying the low-level feature with the previous-level decoded feature and inserting the VAB to obtain the global contextual information. In summary, our method can effectively fuse complementary information between two-modal features and recover the details of salient objects. We conducted extensive experiments on three RGB-T datasets, and the results showed that our method is competitive compared with 15 state-of-the-art methods.

## Figures and Tables

**Table 1:** Quantitative comparisons with the heavy-model-based methods on the RGB-T datasets. Param means the number of parameters. FLOP means floating point operations. FPS means frames per second, which was tested on two types of processors, i.e., professional graphics processing unit GeForce RTX 2080 Ti (GPU) and central processing unit AMD Ryzen 7 5800H @ 3.2 GHz (CPU), respectively. The top three results are marked in red, green, and blue color in each column, respectively. ↑ and ↓ mean a larger value is better and a smaller value is better, respectively.

		Pub.	Param ↓	FLOP ↓	FPS ↑		VT5000				VT1000				VT821			
			**M**	**G**	**CPU**	**GPU**	M↓	$F_{\beta }$↑	$S_{\alpha }$↑	$E_{\xi }$↑	M↓	$F_{\beta }$↑	$S_{\alpha }$↑	$E_{\xi }$↑	M↓	$F_{\beta }$↑	$S_{\alpha }$↑	$E_{\xi }$↑
RGB	BASNet	CVPR_19_	87.1	127.6	0.94	73.0	0.0542	0.762	0.8386	0.878	0.0305	0.8449	0.9086	0.9223	0.0673	0.7335	0.8228	0.8556
	EGNet	ICCV_19_	108.0	156.8	0.93	95.1	0.0511	0.7741	0.853	0.8886	0.0329	0.8474	0.9097	0.923	0.0637	0.7255	0.8301	0.8581
	CPD	CVPR_19_	47.9	17.8	3.97	38.2	0.0465	0.7859	0.8547	0.8964	0.0312	0.8617	0.9072	0.9308	0.0795	0.7173	0.8184	0.8474
RGB-T	ADF	TMM_22_	−	−	−	−	0.0483	0.7775	0.8635	0.891	0.034	0.8458	0.9094	0.9222	0.0766	0.7159	0.8102	0.8443
	MIDD	TIP_21_	52.4	216.7	1.56	36.5	0.0461	0.7876	0.8561	0.8926	0.0293	0.8695	0.9069	0.9353	0.0446	0.8032	0.8712	0.8974
	MMNet	TCSVT_21_	64.1	42.5	1.79	31.1	0.0433	0.7809	0.8618	0.8894	0.0268	0.8626	0.9133	0.932	0.0397	0.7949	0.8731	0.8944
	MIADPD	NP_22_	−	−	−	−	0.0404	0.7925	0.8786	0.8968	0.0251	0.8674	0.9237	0.936	0.0699	0.7398	0.8444	0.8529
	OSRNet	TIM_22_	15.6	42.4	2.29	63.1	0.0399	0.8207	0.8752	0.9108	0.0221	0.8896	0.9258	0.9491	0.0426	0.8114	0.8751	0.9
	ECFFNet	TCSVT_21_	−	−	−	−	0.0376	0.8083	0.8736	0.9123	0.0214	0.8778	0.9224	0.9482	0.0344	0.8117	0.8761	0.9088
	PCNet	MTA_23_	−	−	−	−	0.0363	0.829	0.8749	0.9188	0.021	0.8865	0.932	0.9482	0.0362	0.8193	0.8734	0.9005
	TAGF	EAAI_23_	36.2	115.1	0.87	33.1	0.0359	0.8256	0.8836	0.9162	0.0211	0.8879	0.9264	0.9508	0.0346	0.8205	0.8805	0.9091
	UMINet	VC_23_	−	−	−	−	0.0354	0.8293	0.882	0.922	0.0212	0.8906	0.926	0.9561	0.0542	0.7891	0.8583	0.8866
	APNet	TETCI_21_	30.4	46.6	0.99	36.9	0.0345	0.8221	0.8751	0.9182	0.0213	0.8848	0.9204	0.9515	0.0341	0.8181	0.8669	0.9121
	Our		6.1	1.5	6.5	34.9	0.0321	0.8463	0.8795	0.932	0.0205	0.9016	0.9229	0.9608	0.0311	0.841	0.8776	0.9262

**Table 2:** Quantitative comparisons with the lightweight methods on the RGB-T datasets. The best result is marked in red color in each column. ↑ and ↓ mean a larger value is better and a smaller value is better, respectively.

	Pub.	Param ↓	FLOP ↓	FPS ↑		VT5000				VT1000				VT821			
		**M**	**G**	**CPU**	**GPU**	M↓	$F_{\beta }$↑	$S_{\alpha }$↑	$E_{\xi }$↑	M↓	$F_{\beta }$↑	$S_{\alpha }$↑	$E_{\xi }$↑	M↓	$F_{\beta }$↑	$S_{\alpha }$↑	$E_{\xi }$↑
CSRNet	TCSVT_21_	1.0	4.4	2.7	24.8	0.0417	0.8093	0.8678	0.9068	0.0242	0.8751	0.9184	0.9393	0.0376	0.8289	0.8847	0.9116
LSNet	TIP_23_	4.6	1.2	11.6	51.1	0.0367	0.8269	0.8764	0.9206	0.0224	0.8874	0.9244	0.9528	0.0329	0.8276	0.8777	0.9179
Our		6.1	1.5	6.5	34.9	0.0321	0.8463	0.8795	0.932	0.0205	0.9016	0.9229	0.9608	0.0311	0.841	0.8776	0.9262

**Table 3:** The *t*-test of our method with the compared methods on the RGB-T datasets. For the evaluation metrics IoU and Dice, the right-sided test was performed. The *p*-value is reported in this table. ↑ mean a larger value is better and a smaller value is better, respectively.

	VT5000		VT1000		VT821	
	IoU↑	Dice↑	IoU↑	Dice↑	IoU↑	Dice↑
LSNet	0.7609	0.8411	0.8627	0.9137	0.7665	0.8393
Our	0.7721	0.8531	0.865	0.916	0.7684	0.8439
	0.7728	0.8531	0.863	0.9149	0.7676	0.8424
	0.7718	0.852	0.8649	0.9161	0.7608	0.8357
	0.7738	0.8538	0.8632	0.9151	0.7669	0.8416
	0.771	0.8519	0.8629	0.9141	0.7685	0.8432
	0.7703	0.8512	0.8624	0.9135	0.765	0.8398
*p*-value	1.9 $\times \;10^{-6}$	4.7 $\times \;10^{-7}$	0.0562	0.0154	0.5938	1.1 $\times \;10^{-8}$

**Table 4:** Six sets of experiment results of our method on the RGB-T datasets. ↑ and ↓ mean a larger value is better and a smaller value is better, respectively.

	VT5000				VT1000				VT821			
**No.**	M↓	$F_{\beta }$↑	$S_{\alpha }$↑	$E_{\xi }$↑	M↓	$F_{\beta }$↑	$S_{\alpha }$↑	$E_{\xi }$↑	M↓	$F_{\beta }$↑	$S_{\alpha }$↑	$E_{\xi }$↑
1	0.0321	0.8463	0.8795	0.932	0.0205	0.9016	0.9229	0.9608	0.0311	0.841	0.8776	0.9262
2	0.0325	0.843	0.8797	0.9311	0.0205	0.8978	0.9215	0.9589	0.0312	0.8385	0.8764	0.9251
3	0.0322	0.8451	0.8797	0.9318	0.0199	0.9004	0.9232	0.9608	0.032	0.8384	0.8735	0.9222
4	0.0324	0.8436	0.88	0.9319	0.0203	0.8973	0.9216	0.9591	0.0316	0.8369	0.8761	0.9244
5	0.0331	0.8401	0.8786	0.9299	0.0205	0.8972	0.9214	0.9597	0.0311	0.8361	0.8773	0.9242
6	0.0332	0.8407	0.8781	0.93	0.0205	0.8981	0.9214	0.9595	0.031	0.8369	0.8753	0.9242

**Table 5:** The *t*-test of our method with the compared methods on the RGB-T datasets. For the evaluation metric M, the left-sided test was performed, while for the other three metrics $F_{\beta }$, $S_{\alpha }$, and $E_{\xi }$, the right-sided test was performed. The *p*-value is reported in this table. ↑ and ↓ mean a larger value is better and a smaller value is better, respectively.

	VT5000				VT1000				VT821			
**Compared Method**	M↓	$F_{\beta }$↑	$S_{\alpha }$↑	$E_{\xi }$↑	M↓	$F_{\beta }$↑	$S_{\alpha }$↑	$E_{\xi }$↑	M↓	$F_{\beta }$↑	$S_{\alpha }$↑	$E_{\xi }$↑
BASNet	4.8 × $10^{-10}$	2.5 × $10^{-9}$	2.2 × $10^{-10}$	2.1 × $10^{-10}$	8.4 × $10^{-10}$	4.8 × $10^{-9}$	9.3 × $10^{-8}$	5.5 × $10^{-10}$	1.6 × $10^{-11}$	1.4 × $10^{-10}$	1.9 × $10^{-9}$	2.7 × $10^{-10}$
EGNet	1.0 × $10^{-9}$	5.7 × $10^{-9}$	2.0 × $10^{-9}$	6.2 × $10^{-10}$	2.9 × $10^{-10}$	6.1 × $10^{-9}$	1.4 × $10^{-7}$	6.1 × $10^{-10}$	2.7 × $10^{-11}$	1.0 × $10^{-10}$	3.9 × $10^{-9}$	3.3 × $10^{-10}$
CPD	4.3 × $10^{-9}$	1.4 × $10^{-8}$	2.8 × $10^{-9}$	1.7 × $10^{-9}$	6.0 × $10^{-10}$	3.1 × $10^{-8}$	5.7 × $10^{-8}$	2.0 × $10^{-9}$	3.7 × $10^{-12}$	7.0 × $10^{-11}$	1.3 × $10^{-9}$	1.6 × $10^{-10}$
ADF	2.4 × $10^{-9}$	7.3 × $10^{-9}$	2.5 × $10^{-8}$	8.3 × $10^{-10}$	1.9 × $10^{-10}$	5.2 × $10^{-9}$	1.3 × $10^{-7}$	5.5 × $10^{-10}$	5.0 × $10^{-12}$	6.6 × $10^{-11}$	6.5 × $10^{-10}$	1.3 × $10^{-10}$
MIDD	5.0 × $10^{-9}$	1.7 × $10^{-8}$	3.7 × $10^{-9}$	1.0 × $10^{-9}$	1.6 × $10^{-9}$	1.0 × $10^{-7}$	5.1 × $10^{-8}$	4.6 × $10^{-9}$	2.3 × $10^{-9}$	3.5 × $10^{-8}$	0.0003	2.9 × $10^{-8}$
MMNet	1.6 × $10^{-8}$	9.5 × $10^{-9}$	1.5 × $10^{-8}$	6.9 × $10^{-10}$	8.1 × $10^{-9}$	3.5 × $10^{-8}$	8.0 × $10^{-7}$	2.5 × $10^{-9}$	2.3 × $10^{-8}$	1.2 × $10^{-8}$	0.0024	1.7 × $10^{-8}$
MIADPD	7.7 × $10^{-8}$	2.7 × $10^{-8}$	0.0399	1.8 × $10^{-9}$	3.8 × $10^{-8}$	7.2 × $10^{-8}$	0.9980	5.4 × $10^{-9}$	1.1 × $10^{-11}$	2.0 × $10^{-10}$	2.5 × $10^{-8}$	2.3 × $10^{-10}$
OSRNet	1.1 × $10^{-7}$	1.5 × $10^{-6}$	2.1 × $10^{-5}$	2.5 × $10^{-8}$	5.5 × $10^{-6}$	3.2 × $10^{-5}$	0.9999	2.9 × $10^{-7}$	5.2 × $10^{-9}$	1.4 × $10^{-7}$	0.0932	4.9 × $10^{-8}$
ECFFNet	7.0 × $10^{-7}$	1.7 × $10^{-7}$	4.1 × $10^{-6}$	3.7 × $10^{-8}$	6.9 × $10^{-5}$	5.4 × $10^{-7}$	0.8566	1.9 × $10^{-7}$	3.4 × $10^{-6}$	1.4 × $10^{-7}$	0.5414	4.5 × $10^{-7}$
PCNet	3.1 × $10^{-6}$	1.5 × $10^{-5}$	1.5 × $10^{-5}$	3.0 × $10^{-7}$	0.0007	7.7 × $10^{-6}$	1	1.9 × $10^{-7}$	3.4 × $10^{-7}$	7.8 × $10^{-7}$	0.0038	5.4 × $10^{-8}$
TAGF	5.5 × $10^{-6}$	5.2 × $10^{-6}$	1.5 × $10^{-5}$	1.2 × $10^{-7}$	0.0004	1.4 × $10^{-5}$	0.9999	6.8 × $10^{-5}$	2.5 × $10^{-6}$	1.1 × $10^{-6}$	0.9996	5.0 × $10^{-7}$
UMINet	1.2 × $10^{-5}$	1.7 × $10^{-5}$	0.0001	1.4 × $10^{-6}$	0.0002	5.6 × $10^{-5}$	0.9999	5.4 × $10^{-5}$	1.5 × $10^{-10}$	6.4 × $10^{-9}$	4.5 × $10^{-7}$	5.5 × $10^{-9}$
APNet	7.9 × $10^{-5}$	2.1 × $10^{-6}$	1.9 × $10^{-5}$	2.4 × $10^{-7}$	0.0001	4.0 × $10^{-6}$	0.0025	1.0 × $10^{-6}$	5.6 × $10^{-6}$	5.7 × $10^{-7}$	1.2 × $10^{-5}$	1.5 × $10^{-6}$
CSRNet	3.6 × $10^{-8}$	2.0 × $10^{-7}$	1.2 × $10^{-7}$	1.0 × $10^{-8}$	1.1 × $10^{-7}$	2.9 × $10^{-7}$	6.1 × $10^{-5}$	1.1 × $10^{-8}$	9.7 × $10^{-8}$	2.8 × $10^{-5}$	0.9999	1.2 × $10^{-6}$
LSNet	1.9 × $10^{-6}$	7.6 × $10^{-6}$	0.0001	6.6 × $10^{-7}$	2.5 × $10^{-6}$	1.1 × $10^{-5}$	0.9996	2.4 × $10^{-6}$	9.0 × $10^{-5}$	1.4 × $10^{-5}$	0.9794	3.4 × $10^{-5}$

**Table 6:** Ablation studies of our method on three RGB-T datasets. The best result is marked in red color in each column. ↑ and ↓ mean a larger value is better and a smaller value is better, respectively.

	VT5000				VT1000				VT821			
	M↓	$F_{\beta }$↑	$S_{\alpha }$↑	$E_{\xi }$↑	M↓	$F_{\beta }$↑	$S_{\alpha }$↑	$E_{\xi }$↑	M↓	$F_{\beta }$↑	$S_{\alpha }$↑	$E_{\xi }$↑
*w*/*o* CMIMR	0.0338	0.8321	0.8744	0.9274	0.0222	0.8881	0.9174	0.9556	0.0334	0.8249	0.8682	0.9163
*w*/*o* PDFE	0.0328	0.8396	0.8762	0.9295	0.0211	0.8935	0.92	0.9571	0.033	0.8309	0.8693	0.9182
*w*/*o* IMR	0.0331	0.8394	0.8777	0.9292	0.0208	0.8945	0.9203	0.9577	0.0321	0.8308	0.8712	0.9208
*w* ADF-TMF	0.0329	0.8396	0.8778	0.9309	0.0208	0.8934	0.9189	0.9591	0.0314	0.8368	0.8766	0.9259
*w*/*o* SIGF	0.0334	0.8366	0.8767	0.9287	0.0215	0.8853	0.9159	0.9541	0.0316	0.827	0.8747	0.9207
*w*/*o* SIE	0.0327	0.8405	0.8784	0.93	0.0208	0.8927	0.9202	0.9571	0.0335	0.8308	0.8712	0.9201
*w*/*o* VAB	0.033	0.8392	0.8771	0.9299	0.0208	0.894	0.9199	0.9572	0.0312	0.8327	0.8748	0.9229
*w* ADF-Decoder	0.0328	0.8377	0.8783	0.9299	0.021	0.8941	0.9198	0.9582	0.0319	0.8354	0.8772	0.9238
*w* SIGF-FAM	0.0328	0.8416	0.8795	0.9312	0.0205	0.8965	0.9215	0.9595	0.0316	0.8351	0.8775	0.9231
*w* SIGF-RFB	0.0328	0.8411	0.8794	0.9302	0.0208	0.8966	0.9219	0.9584	0.0328	0.8354	0.8766	0.9221
*w*/*o* IoU	0.0331	0.8344	0.8788	0.9276	0.0222	0.8828	0.9216	0.9488	0.0332	0.8259	0.8764	0.9165
$S^{F}$	0.0327	0.8396	0.8847	0.9289	0.0211	0.8903	0.9269	0.9499	0.0304	0.8353	0.8872	0.9219
$S^{R}$	0.0419	0.7967	0.8578	0.9065	0.0265	0.8727	0.9139	0.9403	0.0427	0.7716	0.8446	0.8914
$S^{T}$	0.0461	0.7608	0.8389	0.8911	0.0354	0.8327	0.8864	0.9204	0.0518	0.745	0.8228	0.8751
$S^{F}$ + $S^{R}$ + $S^{T}$	0.0402	0.7649	0.8774	0.8844	0.0276	0.844	0.9214	0.9216	0.0407	0.7677	0.8793	0.8802
*w* LPW	0.0335	0.8316	0.8818	0.9255	0.0211	0.8861	0.9259	0.9493	0.0311	0.8296	0.8891	0.9199
*w*/*o* AD	0.036	0.8294	0.8778	0.9228	0.0211	0.8902	0.9261	0.9522	0.0334	0.8277	0.8794	0.9198
RGB	0.0419	0.8105	0.8616	0.9115	0.0257	0.8809	0.916	0.9467	0.0543	0.7638	0.8431	0.8939
T	0.044	0.7766	0.8439	0.9007	0.0339	0.8444	0.8884	0.9286	0.0494	0.7595	0.8249	0.8853
Our	0.0321	0.8463	0.8795	0.932	0.0205	0.9016	0.9229	0.9608	0.0311	0.841	0.8776	0.9262

**Table 7:** The *t*-test of our method with ablation experiments on the RGB-T datasets. For the evaluation metric M, the left-sided test was performed. For the other three metrics $F_{\beta }$, $S_{\alpha }$, and $E_{\xi }$, the right-sided test was performed. The *p*-value is reported in this table. ↑ and ↓ mean a larger value is better and a smaller value is better, respectively.

	VT5000				VT1000				VT821			
**Ablation Variant**	M↓	$F_{\beta }$↑	$S_{\alpha }$↑	$E_{\xi }$↑	M↓	$F_{\beta }$↑	$S_{\alpha }$↑	$E_{\xi }$↑	M↓	$F_{\beta }$↑	$S_{\alpha }$↑	$E_{\xi }$↑
*w*/*o* CMIMR	0.0006	5.0 × $10^{-5}$	8.6 × $10^{-6}$	0.0001	4.2 × $10^{-6}$	1.5 × $10^{-5}$	1.8 × $10^{-5}$	2.9 × $10^{-5}$	2.4 × $10^{-5}$	4.6 × $10^{-6}$	2.5 × $10^{-5}$	1.2 × $10^{-5}$
*w*/*o* PDFE	0.1514	0.0080	8.2 × $10^{-5}$	0.0045	0.0004	0.0005	0.0010	0.0002	6.7 × $10^{-5}$	9.2 × $10^{-5}$	5.3 × $10^{-5}$	4.3 × $10^{-5}$
*w*/*o* IMR	0.0204	0.0064	0.0018	0.0022	0.0036	0.0012	0.0019	0.0008	0.0024	8.6 × $10^{-5}$	0.0003	0.0006
*w* ADF-TMF	0.0771	0.0080	0.0024	0.3017	0.0036	0.0004	0.0001	0.0461	0.3457	0.0824	0.8023	0.9816
*w*/*o* SIGF	0.0037	0.0006	0.0002	0.0008	4.4 × $10^{-5}$	4.8 × $10^{-6}$	4.6 × $10^{-6}$	6.6 × $10^{-6}$	0.0766	1.1 × $10^{-5}$	0.0402	0.0005
*w*/*o* SIE	0.2818	0.0223	0.0179	0.0178	0.0036	0.0002	0.0015	0.0002	1.9 × $10^{-5}$	8.6 × $10^{-5}$	0.0003	0.0002
*w*/*o* VAB	0.0392	0.0052	0.0004	0.0133	0.0036	0.0007	0.0008	0.0003	0.7808	0.0004	0.0495	0.0199
*w* ADF-Decoder	0.1514	0.0014	0.0123	0.0133	0.0007	0.0008	0.0006	0.0025	0.0080	0.0080	0.9431	0.1634
*w* SIGF-FAM	0.1514	0.0906	0.7613	0.5802	0.1177	0.0151	0.0983	0.2068	0.0766	0.0052	0.9694	0.0312
*w* SIGF-RFB	0.1514	0.0473	0.6604	0.0330	0.0036	0.0177	0.3889	0.0044	0.0001	0.0080	0.8023	0.0040
*w*/*o* IoU	0.0204	0.0002	0.0927	0.0001	4.2 × $10^{-6}$	2.1 × $10^{-6}$	0.1434	2.5 × $10^{-7}$	3.9 × $10^{-5}$	6.8 × $10^{-6}$	0.7131	1.3 × $10^{-5}$
$S^{F}$	0.2817	0.0080	0.9999	0.0012	0.0004	4.7 × $10^{-5}$	0.9999	4.3 × $10^{-7}$	0.999	0.0069	1	0.0029
$S^{R}$	3.2 × $10^{-8}$	4.1 × $10^{-8}$	5.4 × $10^{-9}$	9.6 × $10^{-9}$	1.0 × $10^{-8}$	1.8 × $10^{-7}$	1.1 × $10^{-6}$	1.5 × $10^{-8}$	5.0 × $10^{-9}$	1.4 × $10^{-9}$	2.6 × $10^{-8}$	1.1 × $10^{-8}$
$S^{T}$	5.0 × $10^{-9}$	2.4 × $10^{-9}$	2.3 × $10^{-10}$	8.5 × $10^{-10}$	1.2 × $10^{-10}$	1.7 × $10^{-9}$	7.1 × $10^{-10}$	4.3 × $10^{-10}$	2.6 × $10^{-10}$	2.6 × $10^{-10}$	1.9 × $10^{-9}$	1.5 × $10^{-9}$
$S^{F}$ + $S^{R}$ + $S^{T}$	8.8 × $10^{-8}$	3.0 × $10^{-9}$	0.0008	3.9 × $10^{-10}$	4.5 × $10^{-9}$	4.4 × $10^{-9}$	0.0669	5.0 × $10^{-10}$	1.3 × $10^{-8}$	1.1 × $10^{-9}$	0.9988	2.5 × $10^{-9}$
*w* LPW	0.0023	4.0 × $10^{-5}$	0.9998	1.5 × $10^{-5}$	0.0004	6.5 × $10^{-6}$	0.9999	3.2 × $10^{-7}$	0.8996	4.1 × $10^{-5}$	1	0.0002
*w*/*o* AD	4.7 × $10^{-6}$	1.7 × $10^{-5}$	0.0024	2.1 × $10^{-5}$	0.0004	4.5 × $10^{-6}$	0.9999	1.6 × $10^{-6}$	2.4 × $10^{-5}$	1.5 × $10^{-5}$	0.9987	0.0002
RGB	3.2 × $10^{-8}$	2.4 × $10^{-7}$	1.4 × $10^{-8}$	3.0 × $10^{-8}$	2.1 × $10^{-8}$	1.2 × $10^{-6}$	5.0 × $10^{-6}$	1.1 × $10^{-7}$	1.5 × $10^{-10}$	8.0 × $10^{-10}$	2.1 × $10^{-8}$	1.6 × $10^{-8}$
T	1.2 × $10^{-8}$	6.8 × $10^{-9}$	4.5 × $10^{-10}$	3.3 × $10^{-9}$	2.0 × $10^{-10}$	4.6 × $10^{-9}$	9.4 × $10^{-10}$	1.4 × $10^{-9}$	4.9 × $10^{-10}$	6.1 × $10^{-10}$	2.3 × $10^{-9}$	4.6 × $10^{-9}$

**Table 8:** Quantitative comparisons with 10 methods on the RGB-D datasets. The top three results are marked in red, green, and blue color in each row, respectively. ↑ and ↓ mean a larger value is better and a smaller value is better, respectively.

		S2MA	AFNet	ICNet	PSNet	DANet	DCMF	MoADNet	CFIDNet	HINet	LSNet	Our
NJU2K	M↓	0.0533	0.0533	0.052	0.0485	0.0464	0.0427	0.041	0.038	0.0387	0.0379	0.0367
	$F_{\beta }$↑	0.8646	0.8672	0.8676	0.8659	0.8763	0.8804	0.8903	0.891	0.896	0.8998	0.901
	$S_{\alpha }$↑	0.8942	0.8801	0.8939	0.8898	0.8969	0.9125	0.9062	0.9141	0.9151	0.9107	0.9021
	$E_{\xi }$↑	0.9163	0.9188	0.9127	0.9125	0.926	0.9246	0.9339	0.9289	0.9385	0.9401	0.9447
NLPR	M↓	0.03	0.033	0.0284	0.0287	0.0285	0.029	0.0274	0.0258	0.0259	0.0244	0.0242
	$F_{\beta }$↑	0.8479	0.8203	0.865	0.8838	0.8662	0.849	0.8664	0.8803	0.8725	0.8824	0.8917
	$S_{\alpha }$↑	0.9145	0.8994	0.9215	0.9061	0.9137	0.921	0.9148	0.921	0.9212	0.9169	0.9136
	$E_{\xi }$↑	0.9407	0.9306	0.9435	0.9457	0.9478	0.9381	0.9448	0.95	0.9491	0.9554	0.9564
DUT	M↓	0.044	−	0.0722	−	0.0467	0.0351	0.0313	−	−	−	0.0332
	$F_{\beta }$↑	0.8847	−	0.8298	−	0.8836	0.9057	0.9214	−	−	−	0.9212
	$S_{\alpha }$↑	0.903	−	0.8524	−	0.8894	0.9279	0.9269	−	−	−	0.9154
	$E_{\xi }$↑	0.9349	−	0.9012	−	0.929	0.9505	0.9589	−	−	−	0.9531
SIP	M↓	−	−	0.0697	−	0.054	−	0.0585	0.0603	0.0658	0.0492	0.0521
	$F_{\beta }$↑	−	−	0.8334	−	0.8615	−	0.846	0.8565	0.8434	0.8819	0.8805
	$S_{\alpha }$↑	−	−	0.8527	−	0.8771	−	0.8648	0.8632	0.8552	0.8844	0.8709
	$E_{\xi }$↑	−	−	0.899	−	0.9167	−	0.9102	0.9058	0.899	0.9271	0.9178
STERE1000	M↓	0.0508	0.0472	0.0447	0.0521	0.0476	0.0427	0.0424	0.0427	0.049	0.0543	0.0439
	$F_{\beta }$↑	0.8545	0.8718	0.8642	0.8522	0.8581	0.8659	0.8666	0.8789	0.8586	0.8542	0.874
	$S_{\alpha }$↑	0.8904	0.8914	0.9025	0.8678	0.8922	0.9097	0.8989	0.9012	0.8919	0.8707	0.8822
	$E_{\xi }$↑	0.9254	0.9337	0.9256	0.9066	0.9263	0.9298	0.9343	0.9325	0.9273	0.9194	0.9364

**Table 9:** Hypothesis test of our method with the compared methods on the RGB-D datasets. The *t*-test was used in our hypothesis test. For the evaluation metric M, the left-sided test was performed. For other three metrics $F_{\beta }$, $S_{\alpha }$, and $E_{\xi }$, the right-sided test was performed. The *p*-value is reported in this table. ↑ and ↓ mean a larger value is better and a smaller value is better, respectively.

		Our						S2MA	AFNet	ICNet	PSNet	DANet	DCMF	MoADNet	CFIDNet	HINet	LSNet
NJU2K	M↓	0.0367	0.037	0.0363	0.0359	0.0361	0.0362	8.8 × $10^{-10}$	8.8 × $10^{-10}$	1.3 × $10^{-9}$	4.6 × $10^{-9}$	1.2 × $10^{-8}$	1.2 × $10^{-7}$	5.6 × $10^{-7}$	9.4 × $10^{-5}$	1.7 × $10^{-5}$	0.0001
	$F_{\beta }$↑	0.901	0.9013	0.9013	0.9028	0.9034	0.9035	2.8 × $10^{-9}$	4.0 × $10^{-9}$	4.2 × $10^{-9}$	3.3 × $10^{-9}$	1.8 × $10^{-8}$	4.3 × $10^{-8}$	8.6 × $10^{-7}$	1.2 × $10^{-6}$	2.1 × $10^{-5}$	0.0018
	$S_{\alpha }$↑	0.9021	0.9018	0.9027	0.9039	0.9034	0.9034	8.1 × $10^{-7}$	6.6 × $10^{-9}$	6.9 × $10^{-7}$	1.1 × $10^{-7}$	5.1 × $10^{-6}$	1	0.9999	1	1	1
	$E_{\xi }$↑	0.9447	0.9442	0.9447	0.9451	0.945	0.945	2.3 × $10^{-11}$	3.6 × $10^{-11}$	1.3 × $10^{-11}$	1.2 × $10^{-11}$	1.8 × $10^{-10}$	1.3 × $10^{-10}$	2.8 × $10^{-9}$	4.2 × $10^{-10}$	4.4 × $10^{-8}$	1.9 × $10^{-7}$
NLPR	M↓	0.0242	0.0245	0.0247	0.0245	0.0243	0.0246	4.6 × $10^{-9}$	5.3 × $10^{-10}$	2.5 × $10^{-8}$	1.8 × $10^{-8}$	2.2 × $10^{-8}$	1.3 × $10^{-8}$	1.1 × $10^{-7}$	5.5 × $10^{-6}$	3.9 × $10^{-6}$	0.7897
	$F_{\beta }$↑	0.8917	0.8888	0.8898	0.8922	0.8925	0.8927	7.4 × $10^{-9}$	6.3 × $10^{-10}$	9.1 × $10^{-8}$	4.5 × $10^{-5}$	1.1 × $10^{-7}$	8.4 × $10^{-9}$	1.2 × $10^{-7}$	6.9 × $10^{-6}$	4.8 × $10^{-7}$	2.0 × $10^{-5}$
	$S_{\alpha }$↑	0.9136	0.9119	0.9127	0.9129	0.913	0.9122	0.9996	2.1 × $10^{-8}$	1	6.8 × $10^{-7}$	0.9948	1	0.9998	1	1	0.9999
	$E_{\xi }$↑	0.9564	0.9548	0.9551	0.9556	0.9561	0.9557	1.1 × $10^{-8}$	8.4 × $10^{-10}$	3.1 × $10^{-8}$	8.5 × $10^{-8}$	2.8 × $10^{-7}$	5.0 × $10^{-9}$	5.5 × $10^{-8}$	1.4 × $10^{-6}$	6.9 × $10^{-7}$	0.2078
DUT	M↓	0.0332	0.0331	0.0321	0.0324	0.0321	0.0326	1.4 × $10^{-8}$	-	2.8 × $10^{-11}$	-	4.8 × $10^{-9}$	2.5 × $10^{-5}$	0.9994	-	-	-
	$F_{\beta }$↑	0.9212	0.9192	0.9224	0.9214	0.9229	0.9205	6.8 × $10^{-9}$	-	7.0 × $10^{-11}$	-	5.9 × $10^{-9}$	4.8 × $10^{-7}$	0.5922	-	-	-
	$S_{\alpha }$↑	0.9154	0.9142	0.9156	0.9145	0.9156	0.9141	8.1 × $10^{-8}$	-	2.0 × $10^{-11}$	-	1.8 × $10^{-9}$	1	1	-	-	-
	$E_{\xi }$↑	0.9531	0.9546	0.9553	0.9544	0.9558	0.9545	2.4 × $10^{-8}$	-	1.6 × $10^{-10}$	-	6.4 × $10^{-9}$	5.5 × $10^{-5}$	0.9999	-	-	-
SIP	M↓	0.0521	0.0507	0.0553	0.0536	0.0534	0.0542	-	-	9.6 × $10^{-7}$	-	0.1443	-	0.0002	6.1 × $10^{-5}$	3.7 × $10^{-6}$	0.9991
	$F_{\beta }$↑	0.8805	0.8855	0.8759	0.8781	0.8798	0.8773	-	-	2.2 × $10^{-7}$	-	2.3 × $10^{-5}$	-	1.1 × $10^{-6}$	7.0 × $10^{-6}$	7.5 × $10^{-7}$	0.9280
	$S_{\alpha }$↑	0.8709	0.8759	0.8661	0.8693	0.8697	0.868	-	-	2.7 × $10^{-5}$	-	0.9983	-	0.0062	0.0021	5.7 × $10^{-5}$	0.9999
	$E_{\xi }$↑	0.9178	0.9211	0.9113	0.9155	0.915	0.9133	-	-	3.7 × $10^{-5}$	-	0.7525	-	0.0058	0.0005	3.7 × $10^{-5}$	0.9998
STERE1000	M↓	0.0439	0.0453	0.0443	0.0441	0.0445	0.0444	2.7 × $10^{-7}$	1.6 × $10^{-5}$	0.1052	1.1 × $10^{-7}$	8.3 × $10^{-6}$	0.9998	0.9999	0.9998	1.4 × $10^{-6}$	3.0 × $10^{-8}$
	$F_{\beta }$↑	0.874	0.8691	0.8728	0.8747	0.8758	0.877	6.1 × $10^{-6}$	0.0608	0.0002	3.5 × $10^{-6}$	1.7 × $10^{-5}$	0.0004	0.0007	0.9966	1.9 × $10^{-5}$	5.6 × $10^{-6}$
	$S_{\alpha }$↑	0.8822	0.88	0.8807	0.8818	0.8809	0.8812	1	1	1	7.8 × $10^{-8}$	1	1	1	1	1	2.6 × $10^{-7}$
	$E_{\xi }$↑	0.9364	0.9352	0.9353	0.9363	0.9359	0.9365	4.9 × $10^{-8}$	0.0001	5.4 × $10^{-8}$	2.9 × $10^{-10}$	7.6 × $10^{-8}$	7.2 × $10^{-7}$	0.0004	1.3 × $10^{-5}$	1.3 × $10^{-7}$	5.1 × $10^{-9}$

## Data Availability

The experiment results in this article are publicly available in this repository: https://github.com/lvchengtao/CMIMR (accessed on 28 January 2024).
